# A pore-occluding phenylalanine gate prevents ion slippage through plant ammonium transporters

**DOI:** 10.1038/s41598-019-53333-9

**Published:** 2019-11-14

**Authors:** Pascal Ganz, Robin Mink, Toyosi Ijato, Romano Porras-Murillo, Uwe Ludewig, Benjamin Neuhäuser

**Affiliations:** Institute of Crop Science, Nutritional Crop Physiology, University of Hohenheim, Fruwirthstr. 20, 70593 Stuttgart, Germany

**Keywords:** Ion channels, Plant molecular biology

## Abstract

Throughout all kingdoms of life, highly conserved transport proteins mediate the passage of ammonium across membranes. These transporters share a high homology and a common pore structure. Whether NH_3_, NH_4_^+^ or NH_3_ + H^+^ is the molecularly transported substrate, still remains unclear for distinct proteins. High-resolution protein structures of several ammonium transporters suggested two conserved pore domains, an external NH_4_^+^ recruitment site and a pore-occluding twin phenylalanine gate, to take over a crucial role in substrate determination and selectivity. Here, we show that while the external recruitment site seems essential for AtAMT1;2 function, single mutants of the double phenylalanine gate were not reduced in their ammonium transport capacity. Despite an unchanged ammonium transport rate, a single mutant of the inner phenylalanine showed reduced N-isotope selection that was proposed to be associated with ammonium deprotonation during transport. Even though ammonium might pass the mutant AMT pore in the ionic form, the transporter still excluded potassium ions from being transported. Our results, highlight the importance of the twin phenylalanine gate in blocking uncontrolled ammonium ion flux.

## Introduction

Ammonium is one of the major inorganic nitrogen sources for plants. In *Arabidopsis thaliana*, other plants and organisms from all kingdoms of life, ammonium is transported across membranes by proteins of the AMT (AMmonium Transporter) / Rh (Rhesus protein)/Mep (Methylammonium permease) family.

The uptake of ammonium (here referring to the sum of NH_3_ and NH_4_^+^) was comprehensively studied in model organisms, where it has been shown that ammonium can be taken up against its concentration gradient. This results in a significant accumulation of ammonium within cells^1–4^. At acidic pH, the NH_4_^+^ ion is by orders of magnitude more abundant than NH_3_ (pK_a_ = 9.25). The uptake of ammonium by plants is biphasic and is constituted of a high-affinity transport system component (HATS) and a low-affinity transport system component (LATS). AMT proteins build the plant ammonium HATS, while the molecular identity of the ammonium LATS remains elusive^5^. Plant AMTs further split into transport proteins conducting net ammonium (NH_3_ + H^+^) transport (AMT1s) and AMT2-like transporters, conducting NH_3_^6–10^.

Protein structures of AMT proteins from bacteria and archaea allow comparisons of the pore structures of different AMTs^11–13^. It is striking that otherwise diverse AMTs like AtAMT1;2 and EcAmtB (*E*. *coli* Ammonium transporter B) share very high identity in their pore-lining residues^7,9^. A close look at the pore structure of EcAmtB shows a conserved outer recruitment site for NH_4_^+^ with the amino acid residue Asp_160_ serving as countercharge to stabilize the recruited NH_4_^+^^14–17^. This aspartic acid residue was further proposed to hold an important function in stabilizing the AMT protein structure^11,12,14,15,18^. A conserved serine^11,16,19^ and alanine^17^ appear to be involved in replacing the water in the hydration shell of NH_4_^+^ before it enters the AMT pore^12,15,20^.

After the recruitment of NH_4_^+^ at the external vestibule, deprotonation to ammonia was proposed by studies on structural constraints and molecular dynamics simulation on the ammonium transport mechanism of the AMT/Rh/Mep proteins. Different sites of deprotonation were suggested^14–16,18,20–28^. Directly adjacent to the NH_4_^+^ recruitment site, a pore-occluding double phenylalanine gate appears to block further transport of NH_4_^+^ into the pore. Some computational studies proposed that deprotonation of the substrate might occur during the translocation from the external vestibule to the inside of the pore, while passing the phenylalanine gate^15,16,18,20,26^. Other studies suggested that the ammonium ion is guided into the channel lumen, crossing the phenylalanine gate before it is deprotonated at the position of a conserved twin-His structure^14,21^. Mutational studies of the two phenylalanines in EcAmtB highlighted their importance in the ammonium transport mechanism. Exchange of F_107_ by alanine and leucine yielded a completely active transporter^19,29^, but the second Phe was important for the transport function. Replacement of the second phenylalanine by leucine strongly reduced ammonium uptake, as well as methylammonium transport^19,29^. Change of this inner phenylalanine to alanine inactivated the transporter. Most double mutants of the phenylalanines in EcAmtB e.g. the F_107_A F_215_A mutant were completely inactive in ammonium transport^19^. This inactive double phenylalanine to alanine mutant potentially mimics the configuration of an “open” transport pathway. Molecular dynamics simulations of ammonium transport by this mutant proposed that NH_4_^+^ is stabilized by the protein backbone at the position of the phenylalanine gate. As a consequence, ammonium deprotonation during its transportation occurs beyond the Phe-gate^19^ possibly at the adjacent conserved twin-His structure. This implies that the phenylalanine gate might not directly be involved in substrate deprotonation.

Ammonium transport in mutants lacking both phenylalanines could partially be rescued by introducing another mutation, W_148_L^19,29^. This rescue was not due to charge stabilization in the external vestibule, as W_148_ was computed to be only of minor importance for stabilizing the ammonium charge in the binding site^14^. Introduction of the W_148_L mutation strongly increased methylammonium transport in AmtB. Mutations in the corresponding residue in an AMT1;1 from tomato reduced transport rates for NH_4_^+^ and MeA^6^. In consequence, it was argued that the double phenylalanine gate is dispensable for transport by EcAmtB^29^.

When addressing the function of the Phe-gate in the human RhCG transporter, already a mutation of one of the Phe residues resulted in a dramatic activity loss. Interestingly, the double mutant, which is non-functional in EcAmtB, was active in RhCG^30^. The combined results point to a species-specific importance of the external vestibule and the Phe-gate in distinct ammonium transporters.

The Phe-gate function has not yet been investigated for the functionally unique AMT1 net ammonium transporters from plants. We hypothesized that the Phe-gate is dispensable for function. Here we analyzed the role of the Phe-gate and the conserved residues in the external vestibule in substrate recruitment, deprotonation and selectivity of AtAMT1;2.

## Results

### The function of the NH_4_^+^ recruitment pocket residues is conserved between plant and bacterial transporters

**Figure 1:**
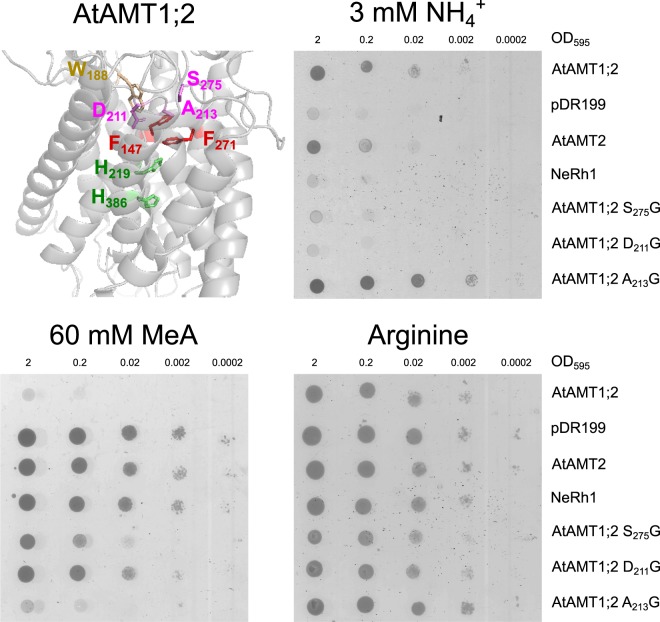
Functional assessment of recruitment pocket mutants by complementation of the *ΔΔΔmep* yeast. Homology model of AtAMT1;2 (top left) based on the ScMEP2 protein structure^37^. The model shows the location and orientation of the amino acid residues investigated in this study (D_211_, A_213_, S_275_ in the recruitment pocket, the Phe gate F_147_ and F_271_, W_188_ and the twin His motif H_219_ and H_386_). *ΔΔΔmep* yeast was transformed with empty vector (pDR199) or pDR199 containing wildtype or mutant AtAMTs as well as NeRh1. The transformed yeast was spotted in 10-fold dilutions beginning with an OD_595_ = 2 on media containing arginine (control = bottom right), ammonium (3 mM = top right) as sole nitrogen source or Arginine with 60 mM MeA (bottom left). Yeast growth on ammonium indicates ammonium transport, while no growth on toxic MeA indicates MeA uptake. Three independent repetitions have been performed, the figure shows one representative experiment.

Multiple studies highlighted three highly conserved amino acid residues to be essential in electrostatic stabilization of the charged substrate in the NH_4_^+^ recruitment pocket^11–13^. Based on a structural alignment and homology modeling, previous studies identified the respective residues in AtAMT1;2^7,10^. Asp_211_, Ala_213_ as well as Ser_275_ (Fig. 1) were proposed to either coordinate the substrate or stabilize the substrate charge within the external vestibule^14–17^. We addressed the importance of these residues in AtAMT1;2 by changing them into glycine residues and tested functionality of the mutants in yeast. AtAMT1;2 or AtAMT2 rescued yeast growth of an ammonium transporter-deficient yeast strain (*ΔΔΔmep*) with ammonium as sole nitrogen source^8,31^. Asp_211_ and Ser_275_ have side chains possibly involved in direct hydrogen bonding with NH_4_^+^. While Asp_211_ was essential for transport activity, the S_275_G mutant showed negligible residual activity (Figs 1; S1). Albeit, the A_213_G mutation did not affect transporter activity.

### The highly conserved pore-occluding Phe-gate is not essential for transport

**Figure 2:**
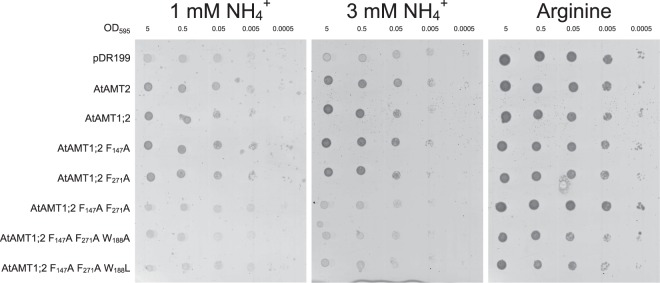
Single Phe mutants complement the growth of the *ΔΔΔmep* yeast on ammonium as the sole nitrogen source. *ΔΔΔmep* yeast was transformed with empty vector (pDR199) or pDR199 containing wildtype or mutant AtAMTs. The transformed yeast was spotted in 10-fold dilutions beginning with an OD_595_ = 5 on media containing arginine (control) or media containing ammonium (1 mM and 3 mM) as the sole nitrogen source. Yeast growth indicates ammonium transport. Four independent repetitions have been performed, the figure shows one representative experiment.

The Phe-gate (F_147_ and F_271_) (Fig. 1) is highly conserved throughout the AMT/Rh/Mep protein family, as it is exhibited by plant AMTs from important model and crop plants like *Arabidopsis thaliana* (AtAMT), *Medicago truncatula* (MtAMT), *Lycopersicon esculentum* (LeAMT), *Oryza sativa* (OsAMT) and *Triticum aestivum* (TaAMT). The neighbouring amino acids residues are highly conserved as well, implying a conserved function in the pore region (Fig. S2). Phenylalanine mutants were analyzed by mutation and growth complementation in *ΔΔΔmep* yeast (Fig. 2).

Single alanine replacements of individual phenylalanines to alanine were tolerated, which in part is in contrast to the situation in EcAmtB, while the simultaneous exchange of both Phe-gate residues abolished ammonium transport (Figs 2; S3). A further amino acid residue, W_188_, was included in the analysis by introducing a W_188_A or W_188_L mutation. Mutation of this residue was able to rescue a Phe double mutant in EcAmtB^29^.

**Figure 3:**
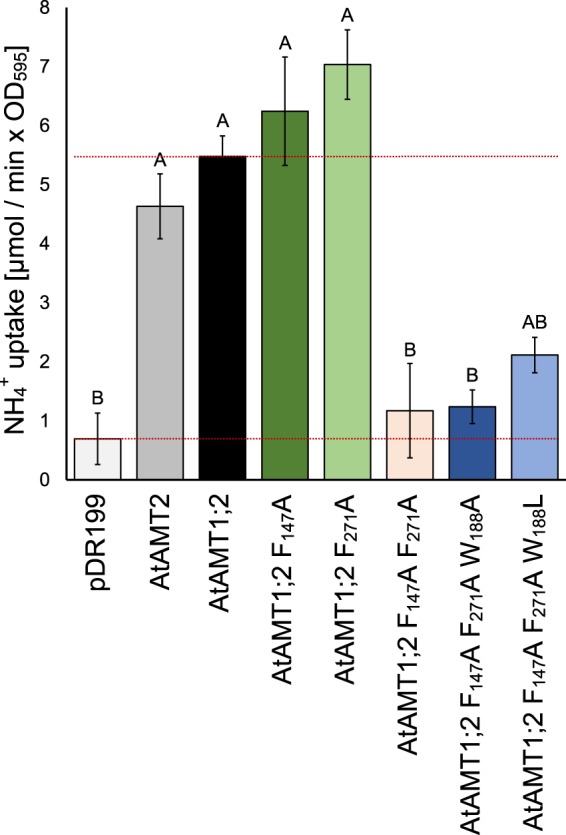
Single mutations in the Phe-gate do not affect the ammonium transport activity of AtAMT1;2. *ΔΔΔmep* yeast expressing the empty vector (pDR199), AtAMT2, AtAMT1;2 or the mutant versions of AtAMT1;2 was grown in liquid media, washed and re-suspended with an OD_595_ = 5 in media containing 3 mM NH_4_^+^. The removal of ammonium from the medium was measured photometrically as described in the methods. The lower line in the graph indicates the background uptake by the empty vector control. The upper line indicates the transport rate of wild type AtAMT1;2. The experiment was performed in four repetitions. Data are shown as means with the error bars indicating the standard error. The data is showing the ammonium uptake by the yeast over time in µM/min x OD_595_. Significant differences with p ≤ 0.05 are indicated by letters.

The transport activity of these mutants was then quantified in liquid culture using an ammonium uptake assay. Negligible residual flux of ammonia into the cells was observed in empty vector controls, while both single Phe mutants retained wildtype AtAMT1;2 transport capacity (Fig. 3). The double mutant did not show significant ammonium uptake, but some residual transport was conferred by the additional W_188_L mutation. Thus, a single Phe is sufficient to maintain plant AMT1;2 ammonium transport function, while double mutants lacking both pore Phe residues were largely inactive.

### Mutation of W_188_ to Ala or Leu established profound MeA transport by the double mutant

**Figure 4:**
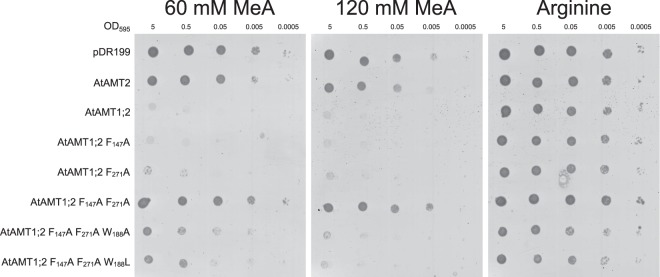
Methylammonium transport by AtAMT1;2 can be partially rescued by combining a W_188_A/L mutation to the double Phe-gate mutant. *ΔΔΔmep* yeast was transformed with empty vector (pDR199) or pDR199 containing wildtype or mutant AtAMTs. The transformed yeast was spotted in 10-fold dilutions beginning with an OD_595_ = 5 on media containing arginine as a nitrogen source and different concentrations of methylammonium (0 mM, 60 mM and 120 mM). Yeast growth indicates low or no methylammonium transport. Four independent repetitions have been performed, the figure shows one representative experiment.

In the AMT1;2 single and double Phe mutants, the ability to transport ammonium directly correlated with the capacity to import the toxic analog MeA, which then impaired yeast growth (Figs 4; S3). While the triple mutants F_147_A F_271_A W_188_A/L failed to transport ammonium (Figs 2, 3), they transported MeA, in contrast to the F_147_A F_271_A double mutant (Fig. 4).

The combined results identify similarity to the situation in EcAmtB and suggest that loss of both Phe in AtAMT1;2 can be compensated by an additional mutation in W_188_ for the substrate MeA but not for ammonium.

### Mutation of Phe_271_ may result in ion slippage through the AtAMT1;2 pore

**Figure 5:**
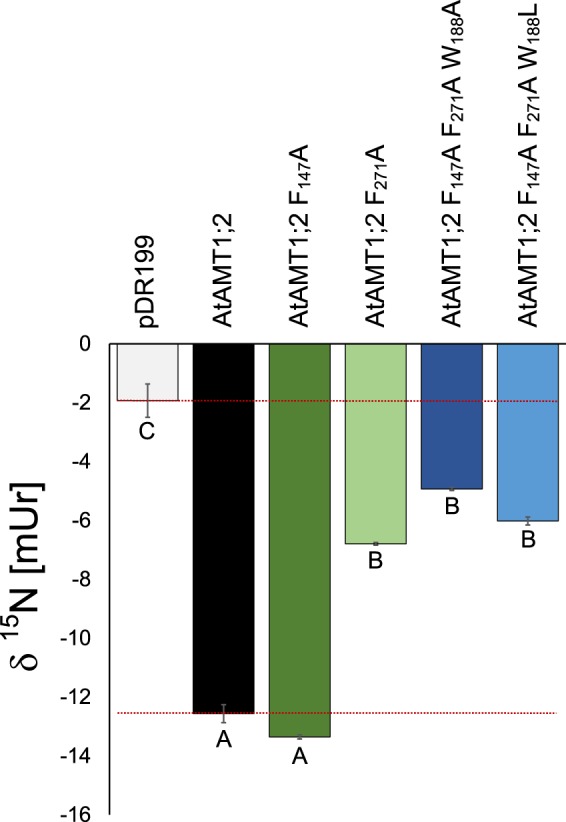
Increase in δ ^15^N values implies a partial loss of deprotonation in F_271_A mutants. *ΔΔΔmep* yeast transfected with wildtype and mutant transporters were grown with 3 mM ammonium chloride in liquid culture and δ ^15^N values were assessed by isotope ratio mass spectrometry. The experiment was performed four times with two technical replications for mass spectrometry. Data are given as means ± SD. Significant different values are indicated by different letters p ≤ 0.01.

In the proposed AMT transport mechanism with NH_4_^+^ recruitment, deprotonation and finally conduction of NH_3_ in the hydrophobic pore^19^, the fate of the proton remained controversial. The basic mechanism of deprotonations was, however, strongly supported by the identification of δ ^15^N shifts caused by ammonium uptake into yeast cells^32^. Discrimination against the heavier isotope was interpreted as a direct indication for deprotonation of the substrate in AMT proteins^32^. The AtAMT1;2 wildtype, as well as the F_147_A single mutant, showed strong isotope discrimination. Interestingly, all variants bearing the F_271_A mutation showed a decreased discrimination of the heavier isotope (Fig. 5), potentially indicating a reduced NH_4_^+^ deprotonation and an increase in ion slippage through the mutant AtAMT1;2 pore.

### Reduced isotope discrimination is not accompanied by a compromised selectivity against K^+^

**Figure 6:**
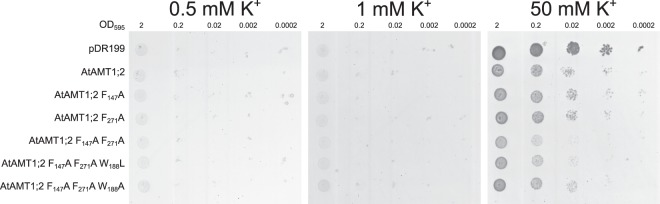
Growth complementation and functionality of AtAMT1;2 Phe-gate mutants in K^+^-uptake deficient WΔ3 yeast. Single, double and triple Phe-gate mutant plasmids were transformed into WΔ3 yeast. 10-fold dilutions of an OD_595_ = 2 culture were spotted on plates containing 0.5, 1 or 50 (control) mM potassium. The experiment was performed three times independently, representative data of one experiment are shown.

Deprotonation of the substrate elegantly explains the high ammonium selectivity of AMT proteins against the similarly-sized K^+^. Therefore, the possibility of K^+^ conduction by the mutants harboring the F_271_A mutation was considered in this study. We hypothesized that if the F_217_A mutant allows NH_4_^+^ ion slippage through the central pore, it may as well allow K^+^ transport. Potassium transport capacity was tested in the WΔ3 yeast strain which lacks two endogenous potassium transporters. This yeast was transformed with all active transporter variants and grown on media containing low mM K^+^ concentrations. Improved growth of the yeast on this medium would indicate facilitated K^+^ transport. None of the transformed transporter variants was able to promote yeast growth (Figs 6, S4). By contrast all constructs slightly impaired growth on high K^+^. This result probably indicates unspecific negative effects by each construct, as the non-functional mutant had the largest impairment relative to the empty plasmid control.

## Discussion

The protein structures of several ammonium transporter proteins from various organisms indicate a conserved pore-occluding twin Phe-gate directly adjacent to the external ammonium recruitment site^14–16,18,20–28^. Based on sequence identity and homology modeling^5,9^, we propose a similar orientation of these two highly conserved phenylalanines in plant AMT1 transporters as in AMT crystal structures. Members of the plant AMT1 family appear to transport net ammonium (as NH_3_ + H^+^)^6,7^, whereas plant AMT2-like proteins seem to transport ammonia after NH_4_^+^ deprotonation^8,9^. While the transport mechanism differs between individual AMT/Mep/Rh proteins (NH_3_, NH_4_^+^ or NH_3_ + H^+^ transport), the pore-lining residues and the basic mechanism with NH_4_^+^ deprotonation after NH_4_^+^ recruitment appear highly conserved^9,10^. The external ammonium recruitment site including Asp_211_, Ala_213_ and Ser_275_ (Fig. 1) and the function of the pore-occluding twin Phe-gate were addressed here. The D_211_G and S_275_G mutations strongly reduced or even impaired AMT activity, in line with a proposed role of substrate acquisition to the external recruitment site or for protein stability^11–20^.

Knowing that the transport mechanism between EcAmtB and AtAMT1s may differ, the Phe-gate might vary in its importance for each individual transporters. In AtAMT1;2, exchange of individual phenylalanines to alanine yielded full transport function (Figs 2–4). For the first (outer) phenylalanine, this is in accordance with previous results in EcAmtB^19,29^. The F_271_A mutant in AtAMT1;2 was completely active while F_215_ of EcAmtB was essential for ammonium and methylammonium transport^19,29^. The double mutants F_147_A F_271_A in AtAMT1;2 and F_107_A F_215_A in EcAmtB were both inactive in ammonium and methylammonium transport. It is still interesting though, that in molecular dynamic simulations of this EcAmtB mutant (mimicking an open phenylalanine gate) the ammonium ion was still stabilized by the protein backbone in the position of the twin-His motif following the Phe-gate^19^. Notably, the introduction of the W_148_L mutation in EcAmtB (W_188_L in AtAMT1;2, respectively) partially rescued the transport function of the EcAmtB F_215_L mutant and the F_107_A F_215_L double mutant, respectively. W_148_ is located in the NH_4_^+^ recruitment site of the transporter and not directly part of the phenylalanine gate. It seems to play a minor role in stabilizing the ammonium charge in the recruitment pocket. The W_188_L mutation will significantly increase the free space within this pocket, possibly facilitating the binding of the more bulky methylammonium^14^. This follows the observations from EcAmtB, in which the individual W_148_L mutation strongly increased methylammonium transport^29^. In AtAMT1;2 triple mutants (F_147_A F_271_A W_188_A or F_147_A F_271_A W_188_L) methylammonium transport was also restored (Fig. 4). Replacements in W_188_, however, did not restore ammonium transport in the double mutant (Figs 2 and 3).

Isotope fractionation during ammonium transport into yeast cells had been proposed to result from easier deprotonation of the lighter N isotope substrate. Ultimately this would result in a higher abundance of the lighter isotope in total assimilated yeast N^32^. The isotope fractionation was reduced by the F_271_ mutation, despite an unchanged transport rate, potentially indicating a partially lacking substrate deprotonation (Fig. 5). These results indicate an important role of F_271_ in the deprotonation mechanism and thus selection against uncontrolled ion flux. Although the Phe-gate was suggested not to be directly involved in deprotonation^19^, F_271_ might be important for stabilizing NH_4_^+^ or its charge during deprotonation. Deprotonation is then likely fulfilled by the adjacent twin-His motif. If slippage of NH_4_^+^ ions occurs, one may expect that ions of similar charge and size, such as K^+^ are transported. However, none of the mutants or the wild type facilitated potassium uptake (Fig. 6). Instead, expression of all mutants impaired growth of the yeast on high K^+^, compared to the empty plasmid control, pointing to a general fitness decrease by AMTs in this yeast strain. Interestingly, the non-functional F_147_A F_271_A mutant was most inhibitory, showing that the transport function of the protein was not the cause of growth impairment.

Taken together, single mutations of the phenylalanines did not affect the transport activity of AtAMT1;2. Mutation of the inner Phe residue resulted in a partial loss of the AMT1;2 mediated isotope fractionation, indicating a partial loss of deprotonation. This as well indicates that the substrate is still protonated at the position of the phenylalanine gate, which would contradict a mechanism of ammonium deprotonation in the external vestibule^15,16,18,20,26^. While deprotonation was partially lost in the mutants, all transporter versions were still selective against potassium.

**Figure 7:**
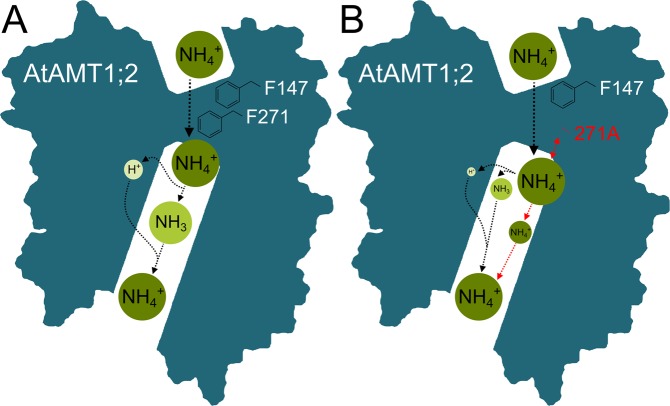
Schematic model of the transport mechanism in the AtAMT1;2 wildtype and the F_271_A mutant. (**A**) In the wildtype transporter, NH_4_^+^ passes the pore and is deprotonated after crossing about 40% of the membrane electric field. On the cytoplasmic side, it is re-protonated to NH_4_^+^ by a co-transported proton. (**B**) In the F_271_A mutant, the transport – deprotonation coupling is partially lost. NH_4_^+^ might directly slip through the AMT pore (red arrows). The red bidirectional arrow indicates reduced coordination of the ammonium ion. This possible loss of ion stabilization might reduce deprotonation efficiency. The schematic model is based on a homology model of AtAMT1;2^7^. The structural fitting for the homology model was done using the crystal structures of AfAMT-1^38^ and EcAmtB^11,12^.

In conclusion, not both Phe of the pore-occluding Phe-gate in the electrogenic AtAMT1;2 can simultaneously be replaced. While selective transport depends on substrate deprotonation^32^ (Fig. 7A), especially the inner Phe residue avoids NH_4_^+^ ion slippage through the pore. Mutation of F_271_ may partially allow NH_4_^+^ ions to pass the pore without fractionation into H^+^ and NH_3_ (Fig. 7B). The loss of the aromatic structure in the F_271_A mutant might decrease the NH_4_^+^ coordination in this position and thereby reduce the deprotonation dependence. Mutation of W_188_ provides a pore recruitment constitution that improves methylammonium entrance to the pore. Therefore, W_188_ recruits NH_4_^+^, but prevents methylammonium entry into the AMT pore.

## Material and Methods

### Plasmid constructs

Briefly, the open reading frames of AtAMT1;2 (At1g64780), AtAMT2 (At2g38290) and NeRh1 (gi_30248465) were amplified from genomic DNA or cDNA of *Arabidopsis thaliana* Col-0 wildtype and cDNA of *Nitrosomonas europaea* respectively. Using specific restriction enzymes they were then subcloned into the yeast expression vector pDR199^7,9,31^. The mutant constructs were based on this original construct. To introduce the mutations, forward and reverse mismatch primers were designed and mutant constructs were produced by mutagenesis PCR using S7 Fusion High-Fidelity DNA Polymerase (Biozym Scientific, Hessisch Oldendorf, Germany). Template DNA was digested by DpnI. To verify the mutations and exclude other mutations the constructs were Sanger sequenced (Eurofins Genomics, Ebersberg, Germany).

### Yeast transformation

The plasmids containing the respective open reading frames were heat shock-transformed in the ura^-^ ammonium transporter defective yeast strain (31019b; *ΔΔΔmep*)^33^ and the potassium transporter defective *Saccharomyces cerevisiae* strain W∆3 (MATa, ade2, ura3, trp1, trk1Δ::LEU2, trk2Δ::HIS3)^34^. Selection for transformed yeast was done on solid arginine media (20 g × l^−1^ Agar, 1.7 g × l^−1^ YNB w/o amino acids and ammonium sulfate (Difco), supplemented with 20 g × l^−1^ glucose and 1 g × l^−1^ arginine (Arg) as nitrogen source) or on Arg phosphate minimal media^35^ containing 100 mM K^+^.

### Yeast growth assays

Yeast was grown in liquid Arg medium until OD_595_ (optical density at 595 nm) reached 0.6–0.8. Cells were harvested, washed three times and resuspended in water to a final OD_595_ of 2 or 5. 10 µl of these cells and 10-fold dilutions were spotted on Arg medium with or without MeA (60 mM or 120 mM), or media containing no Arg, but 1 mM or 3 mM NH_4_Cl as sole nitrogen source. For growth on selective potassium media, yeast was spotted as 10 fold dilutions of OD_595_ of 2, on Arg phosphate minimal media containing different concentrations of K^+^. Pictures were taken after 3 to 5 days of growth, the experiments were performed in three or four independent repetitions.

### Ammonium uptake assay

To determine ammonium uptake rates of the *ΔΔΔmep* yeast expressing the different ammonium transporter constructs, an ammonium uptake assay was conducted. Cells were grown overnight in 50 ml Arg medium supplemented with 1 g × l^−1^ arginine at 28 °C, washed three times in water, and resuspended in Arg medium with 3 mM ammonium chloride at OD_595_ of 3. The cultures were incubated with shaking (200 rpm) at 28 °C in a 30 ml volume and 500 µl samples were taken every 30 min until 3 h. The cells were pelleted and 300 µl supernatant was separated from the pellet and stored at 4 °C. 40 µl of the supernatant was added to 760 µl OPA (o-phthaldialdehyde) solution (540 mg o-phthaldialdehyde, 10 ml ethanol, 50 µl β-mercaptoethanol, 0.2 M phosphate buffer, pH 7.3 ad 100 ml) to quantify the remaining ammonium^36^. After 20 min of incubation in the dark, the extinction at 420 nm was measured. As a reference, 760 µl OPA plus 40 µl water was used. The system was calibrated with ammonium chloride concentrations from 0 to 5 mM. The experiment was performed in four independent repetitions.

### Change in the natural δ15N

Discrimination of the transporters against the heavy ^15^N isotope was determined in yeast. ∆∆∆*mep* yeast was transformed as described above. Cells were grown overnight in 50 ml SD medium with 1 g × l^−1^ arginine at 28 °C. OD_595_ of the overnight culture was determined and a new 150 ml culture in SD medium with 3 mM NH_4_^+^ as sole nitrogen source was inoculated with OD_595_ 0.01. Cultures were incubated at 28 °C with shaking at 120 rpm. Cultures were harvested when OD_595_ reached approx. 0.3. Cells were washed two times, pelleted, freeze-dried and δ ^15^N ratios were determined by isotope ratio mass spectrometry. For IRMS analysis approx. 0.5 mg of yeast or standard material war balanced into tin cups (HEKAtech, Wegberg, Germany). Standard material used was USGS41, L-glutamic acid with δ ^15^ = 47.6‰ air N_2_. For measurement an element analyzer (EuroVector, HEKAtech, Wegberg, Germany) coupled to a mass spectrometer (Delta plus Advantage, Thermo Scientific, Bremen, Germany) was used. δ15 values are calculated vs. Air-N_2_ using the following equation: δ ‰ = (Isotope ratio of sample/Isotope ratio of air - 1) × 1000. Data are given as means from two independent experiments with two biological replicates each ± SD. Significance was calculated by one-way ANOVA followed by a Tukey HSD test.

## Supplementary information

Supplemental Information

## Data Availability

All data are part of the manuscript.
