# Supplementary material for: A pore-occluding phenylalanine gate prevents ion slippage through plant ammonium transporters
Source: Sci Rep. 2019 Nov 14;9:16765. doi: 10.1038/s41598-019-53333-9 (PMC6856177; doi:10.1038/s41598-019-53333-9)
Supplement: Supplementary file 1 — Supplemental Information [file 41598_2019_53333_MOESM1_ESM.pdf]

Supplemental Information

A pore-occluding phenylalanine gate prevents ion slippage through plant ammonium transporters

Pascal Ganz, Robin Mink, Toyosi Ijato, Romano Porras-Murillo, Uwe Ludewig & Benjamin Neuhäuser*

Institute of Crop Science, Nutritional Crop Physiology, University of Hohenheim, Fruwirthstr. 20, 70593 Stuttgart / Germany

*Corresponding author:
Benjamin Neuhäuser
Institute of Crop Science, Nutritional Crop Physiology, University of Hohenheim, Fruwirthstr. 20, 70593 Stuttgart / Germany
Tel.: +49 (0) 711 - 459 22777
Fax.: +49 (0) 711 - 459 23295
email: benjamin.neuhaeuser@uni-hohenheim.de

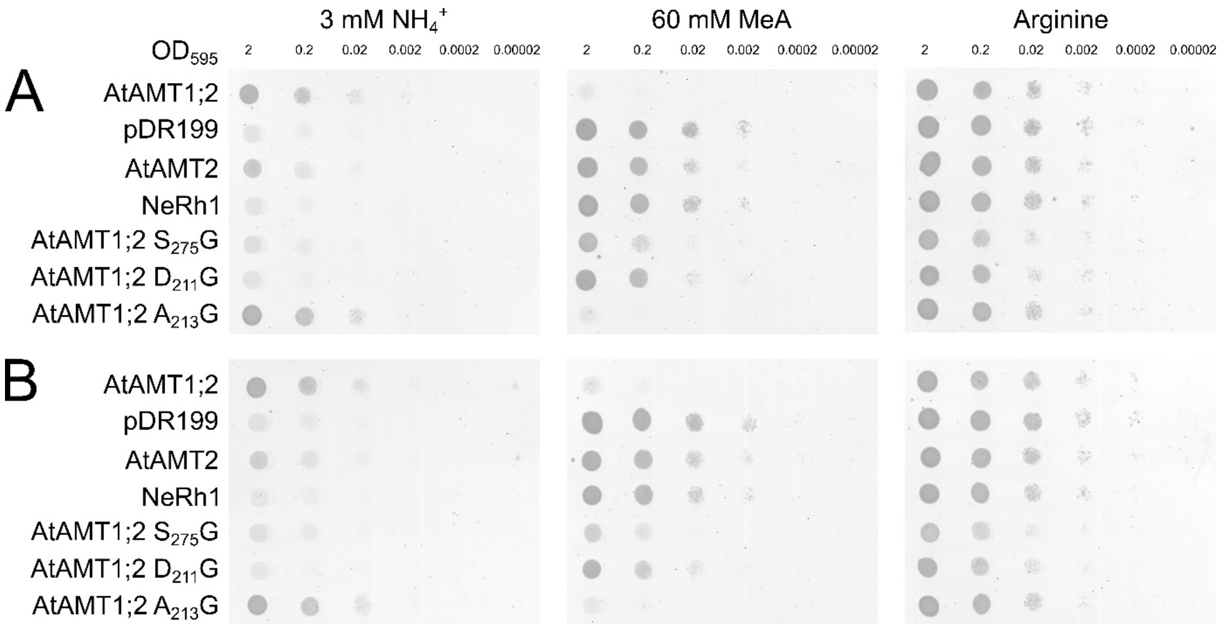

Figure S1: Two additional independent repetitions of the functional assessment of binding pocket mutants by complementation of the *ΔΔΔmep* yeast. *ΔΔΔmep* yeast was transformed with empty vector (pDR199) or pDR199 containing wildtype or mutant AtAMTs as well as NeRh1. The transformed yeast was spotted in 10-fold dilutions beginning with an OD_{595} = 2 on media containing arginine (control = right), ammonium (3 mM = left) as sole nitrogen source or Arginine with 60 mM MeA (middle). A) and B) show two independent repetitions of the experiment shown in Figure 1.

AtAMT1;1	127	...FLYQWAFAIAAAGITSGS...	...LLWFGWYGFNPGSF...
AtAMT1;2	140	...FLYQWAFAIAAAGITSGS...	...LLWFGWYGFNPGSF...
AtAMT1;3	131	...FLYQWAFAIAAAGITSGS...	...LLWFGWYGFNPGSF...
AtAMT1;4	136	...FLYQWTFAIAAAGITSGS...	...LLWFGWYGFNPGSF...
AtAMT1;5	131	...FLYQWAFAIAAAGITSGS...	...LLWFGWYGFNPGSF...
LeAMT1;1	128	...FLYQWAFAIAAAGITSGS...	...LLWFGWYGFNPGSF...
LeAMT1;2	133	...FLYQWAFAIAAAGITSGS...	...LLWFGWYGFNPGSF...
LeAMT1;3	98	...FLYQWAFAIAVAGITSGS...	...LLWFGWFGFNPGSF...
MtAMT1;1	133	...FLYQWAFAIAAAGITSGS...	...MLWFGWYGFNPGSF...
MtAMT1;2	132	...FLFQWAFAIAAAGITSGS...	...LLWFGWYGFNPGSF...
MtAMT1;3	132	...FLYQWAFAIASAGITSGS...	...LLWFGWYGFNPGSF...
OsAMT1;1	120	...FLFQWAFAIAAAGITSGS...	...LLWFGWYGFNPGSF...
OsAMT1;2	122	...FLFQWAFAIAAAGITSGS...	...LLWFGWYGFNPGSF...
OsAMT1;3	122	...FLFQWAFAIAAAGITSGS...	...LLWFGWFGFNPGSF...
TaAMT1;1 2AL	121	...FLFQWAFAIAAAGITSGS...	...LLWFGWYGFNPGSF...
TaAMT1;1 2BL	121	...FLFQWAFAIAAAGITSGS...	...LLWFGWYGFNPGSF...
TaAMT1;1 2DL	121	...FLFQWAFAIAAAGITSGS...	...LLWFGWYGFNPGSF...
TaAMT1;2 6AL	120	...FLFQWAFAIAAAGITSGS...	...LLWFGWYGFNPGSF...
TaAMT1;2 6BL	120	...FLFQWAFAIAAAGITSGS...	...LLWFGWYGFNPGSF...
TaAMT1;2 6DL	120	...FLFQWAFAIAAAGITSGS...	...LLWFGWYGFNPGSF...

Figure S2: Alignment showing high conservation of the Phe-gate region in plant AMT1s. AMT1 protein sequences from important model and crop plants like *Arabidopsis thaliana* (AtAMT), *Medicago truncatula* (MtAMT), *Lycopersicon esculentum* (LeAMT), *Oryza sativa* (OsAMT) and *Triticum aestivum* (TaAMT) were derived from the public database plants.ensembl.org. Sequences were aligned using the Clustal V method and the conservation of protein sequences around the Phe-gate forming phenylalanine residues is shown.

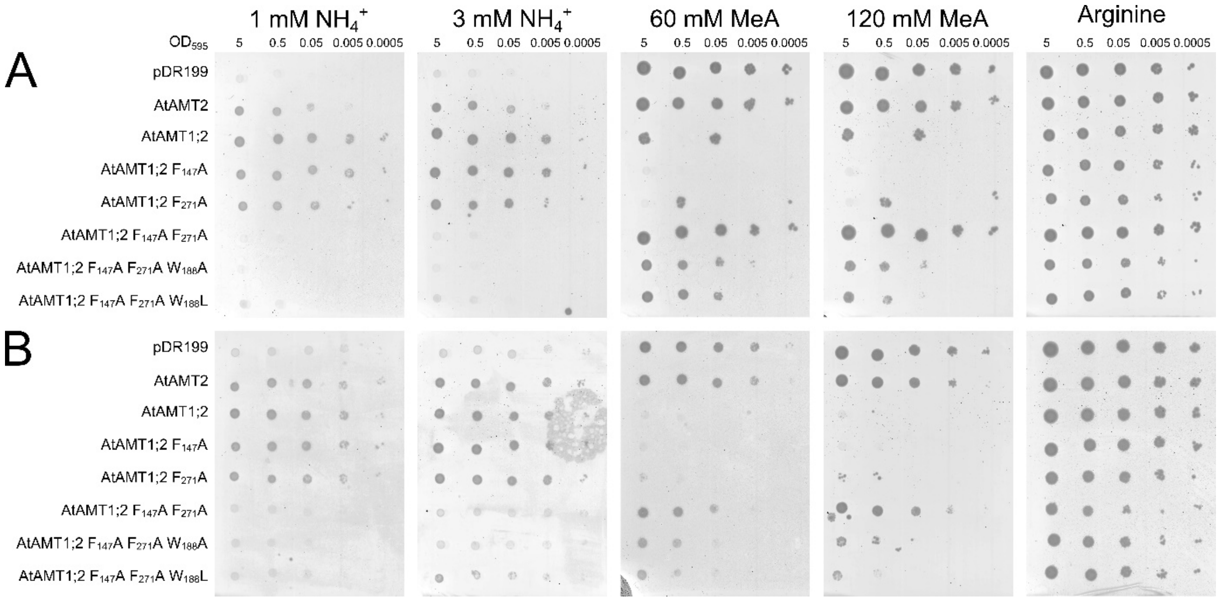

Figure S3: Two additional independent repetitions of the growth of the *ΔΔΔmep* yeast on methylammonium and ammonium as the sole nitrogen source. *ΔΔΔmep* yeast was transformed with empty vector (pDR199) or pDR199 containing wildtype or mutant AtAMTs. The transformed yeast was spotted in 10-fold dilutions beginning with an OD_{595} = 5 on media containing arginine and different concentrations of methylammonium (0 mM, 60 mM and 120 mM) or media containing ammonium (1 mM and 3 mM) as the sole nitrogen source. A) and B) show two additional independent repetitions of the experiments shown in Figure 2 and 4.

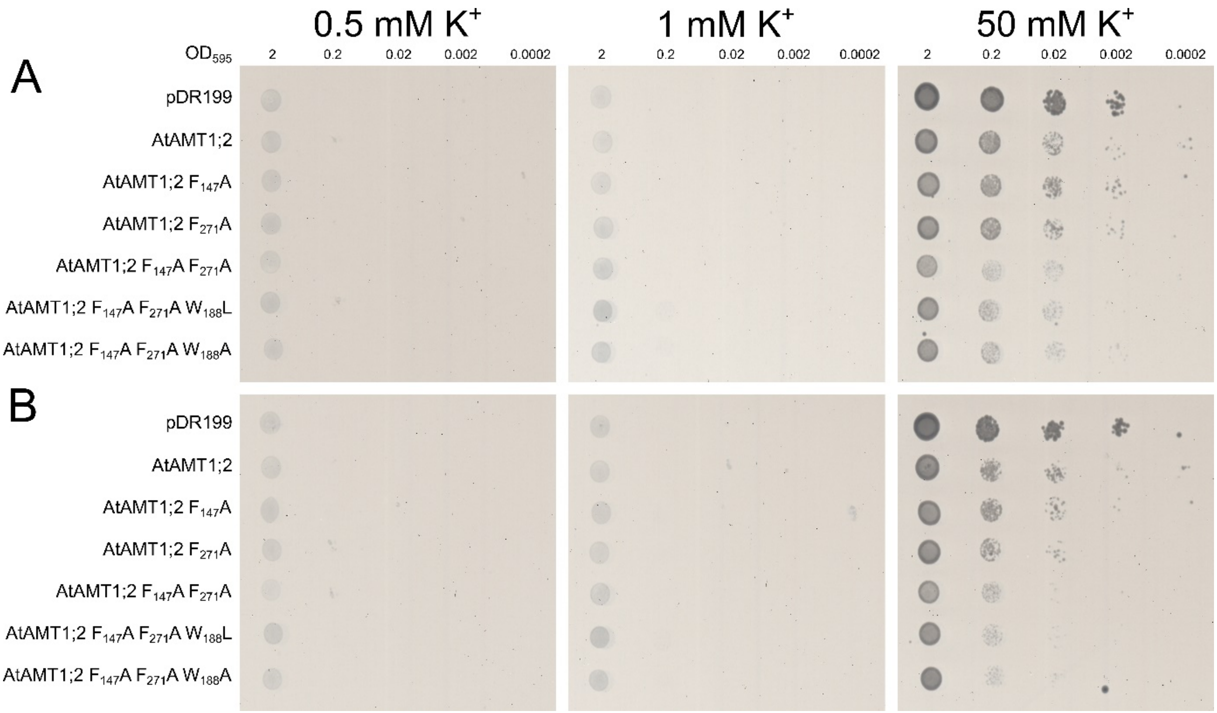

Figure S4: Two independent repetitions of the growth complementation and functionality of AtAMT1;2 Phe-gate mutants in K^+-uptake deficient WΔ3 yeast. Single, double and triple Phe-gate mutants were transformed into WΔ3 yeast. 10-fold dilutions of a OD_{595} = 2 culture were spotted on plates containing 0.5, 1 or 50 (control) mM potassium. A) and B) show two additional independent repetitions of the experiment shown in Figure 6.
